# Long Live Love. The implementation of a school-based sex-education program in the Netherlands

**DOI:** 10.1093/her/cyu021

**Published:** 2014-05-10

**Authors:** Lisette Schutte, Ree M. Meertens, Fraukje E. F. Mevissen, Herman Schaalma, Suzanne Meijer, Gerjo Kok

**Affiliations:** ^1^Department of Work and Social Psychology, Faculty of Psychology and Neuroscience, Maastricht University, PO Box 616, 6200 MD, Maastricht, The Netherlands, ^2^Department of Health Promotion, Nutrition and Toxicology Research Institute Maastricht (NUTRIM) and Care and Public Health Research Institute (Caphri), Maastricht University and ^3^Department of Youth, STI AIDS Netherlands, The Netherlands

## Abstract

Implementation of health education programs is often inadequately considered or not considered at all in planning, developing and evaluating interventions. With the focus being predominantly on the adoption stage, little is known about the factors influencing the implementation and continuation stages of the diffusion process. This study contributes to the understanding of factors that promote or impede each stage of the diffusion process in the school setting using the sex education program Long Live Love (LLL) as an example. A survey integrating different diffusion-related concepts was completed by 130 teachers. Results showed that teacher curriculum-related beliefs were associated with all stages in the diffusion process. Although adoption of LLL was predominantly related to teacher curriculum-related beliefs, implementation completeness and fidelity and continued use of LLL were also enhanced by contextual factors, namely teacher training and interactive context variables (school policy, governing body support and student response), respectively. The results of this study can be used to optimize the adoption, implementation and continuation of school-based (sexual) health promotion programs.

## Introduction

School-based sex-education programs are the primary means by which adolescents in the Netherlands receive information and skills related to safe sex, communication about sex and managing relationships [1]. A multitude of interventions have been developed globally for sex education of youngsters in school [2, 3]. Although sometimes proven effective, other interventions show only short-term or no effects [1, 4]. Besides due to an ineffective content, these inconsistent findings may also be explained by inadequate implementation. Not being completely or correctly implemented can greatly undermine the effectiveness of an intervention [5]. Indeed, the impact of school-based health education programs is often attenuated by inadequate teacher implementation [6]. Implementation is thus a crucial aspect of planning and delivering successful health education programs yet it receives insufficient attention [5, 7, 8].

There are few published accounts of the process of implementation of interventions once they have been formally adopted by schools, particularly in relation to sex education. Little is known about if, how or how well the material is covered. Research conducted in the implementation field has tended to focus primarily on the adoption stage [9]. Considerably less effort has been devoted to determining whether and how new programs are actually used in classrooms after being adopted. The assumption is often made that adoption at the organizational level will result in adoption and implementation at the teacher level. However, program adoption does not guarantee implementation and teachers’ initial attempts will not necessarily result in continued use of the program [5]. Understanding the factors that influence each of these stages is therefore crucial in explaining and improving the effectiveness of school-based sex-education programs specifically or school-based interventions in general.

This study attempts to fill that gap by focusing on all the stages in the diffusion process, providing a holistic explanation of the adoption and implementation behavior of teachers in the school context. The present study addresses the promoting and inhibiting factors of teacher adoption, implementation and maintenance of a Dutch school-based sexual education program called Long Live Love (LLL). The LLL program is one of the most successful, evidence-based programs in the field of school-based sex education in the Netherlands targeted at adolescents (13–15 years) in secondary vocational schools. The effectiveness of previous versions of this program has been largely accredited to the quality and extent of its implementation [10, 11]. In the current study, teacher’s classroom implementation of LLL is evaluated and the determinants of the implementation process are examined.

## Long Live Love

In the Dutch education system, schools and teachers are autonomous in their selection and use of health education programs, without the interference of external authority. Sex education is also provided on a voluntary basis, mostly by biology teachers [12]. LLL is the most widely used evidence-based teacher-delivered program for sexual education in the Netherlands, proving it to be a worthwhile intervention [11]. Over 50% of vocational schools have bought the program [13]. The first version of LLL was developed 24 years ago and was shown to produce desirable student learning outcomes, when correctly applied [11]. Since then the LLL curriculum has been revised three times and another revision is in progress. The last evaluation however was done in 2002 [14]. In order to improve successful implementation of the future LLL curriculum and other school-based (sexual) health promotion programs, an up-to-date evaluation of factors influencing adoption and implementation is necessary.

LLL is a relational and sexual education program composed of 26 learning activities divided over six lessons of one hour each. 22 of these activities are core and 4 are optional. LLL is designed to provide students with communication and negotiation skills to enable safe sex practices. It compromises a teacher’s manual, a student magazine and DVD. The main objective is the prevention of STDs/HIV and unplanned pregnancy [13]. The presented framework will guide the evaluation of the LLL program.

## Research framework

The general outline of the research framework for this study was derived from an integration of Roger’s Diffusion of Innovations theory, the Theory of Planned Behavior (TPB), Social Cognitive Theory and from previous research on innovation in AIDS education in Dutch schools [12, 15].

Roger’s Diffusion Theory [16] describes implementation as a decision-making process consisting of different stages: (1) awareness of an innovation, through spreading information about the program, potential user receiving, requesting and processing information (dissemination), (2) the formation of an intention to buy and use the program (adoption), (3) initial use (implementation) and (4) continued use of the program (maintenance) [5].

The TPB claims that intention is the most important predictor of behavior [17]. According to Paulussen *et al.* [18], intention and behavior in this context can be considered synonymous for adoption and implementation, respectively. ‘Adoption’ thus refers to the intention of teachers to use the curriculum during sexual education. Only once a program has been adopted can it be implemented. ‘Implementation’ of the curriculum refers to performance of the behavior, the actual use of the program. The implementation stage has been defined by two dimensions: quality and quantity. Quantity or extent/completeness is how much of the curriculum is taught; quality or fidelity is the measure in which the program has been implemented as intended by the developers. Different factors influence each stage in the diffusion process.

The framework used in our study included the adoption of a revised version of LLL and the implementation and the continuation stages of the current version of LLL. Adoption of the ‘revised’ LLL program was focused on instead of the ‘current’ LLL program because all respondents included in the study have already adopted the current LLL program and there was more interest in inquiring what factors need to be taken into consideration to promote adoption of the revised LLL. Furthermore, past experience with programs has been found to influence future use of it [12, 19–21].

**Fig. 1.**
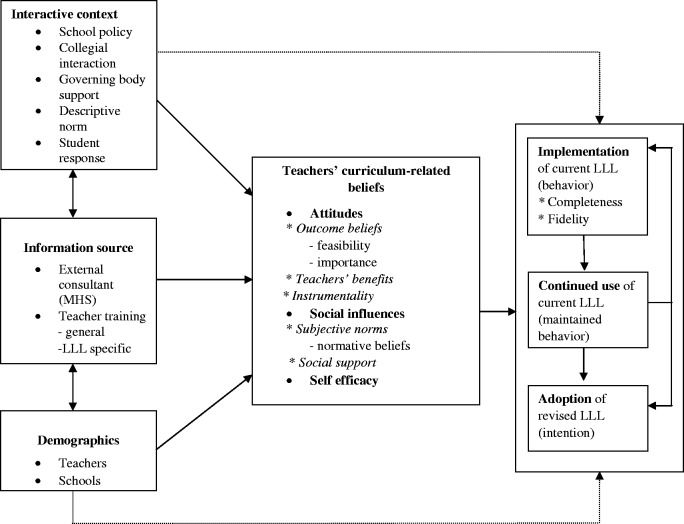
Framework for investigating the correlates of teachers’ LLL implementation-decision process, adapted from Paulussen *et al.*[17].

The framework is presented in Fig. 1. The determinants that influence each of these stages were investigated. These determinants have been placed into four categories: (1) curriculum-related beliefs, (2) interactive context, (3) information sources and (4) demographic variables. Three clusters of curriculum-related beliefs—attitudinal, normative and self-efficacy beliefs—were assumed to affect curriculum adoption, implementation and continuation most directly. These curriculum-related beliefs in turn may be influenced by the other three categories. The interactive context and information source are believed to influence teacher’s adoption decision and implementation and continuation behavior, either directly or indirectly.

### Teacher’s curriculum-related beliefs

Teacher’s classroom implementation is best explained by their curriculum-related beliefs which include their attitudinal, normative and self-efficacy beliefs toward that particular innovation [18, 22, 23]. Perceived importance and feasibility of student learning outcomes (outcome beliefs) are assumed to capture teachers’ attitudes toward classroom sexual education [18]. Also under attitude are ‘teachers’ benefits’ (the personal advantages that the curriculum could have for the teacher) and ‘instrumentality’ (practicality of program use in practice related to how acceptable the intervention is from a practical point of view) [12].

Important individuals might provide normative standards for teachers’ decision to implement a new program [18]. ‘Subjective norms’ are conceptualized as the attributed normative beliefs of important social referents, such as students, colleagues and parents [17]. ‘Social support’ involves the affective and/or instrumental support expected of social referents in the teachers’ environment, namely the governing body, colleagues teaching the same and different subjects and the parent association [23]. ‘Self-efficacy’ refers to one’s perceived ability to perform a particular behavior, in this case, teachers’ ability to implement the LLL curriculum in their classrooms with confidence [24]. Self-efficacy is often found to be a strong predictor of the implementation of curriculum innovations, especially for sex education [12, 25].

Teachers are expected to deliver more of the program (completeness) with higher integrity (fidelity) if they have a more positive judgment of the curriculum (Is it beneficial to use LLL? Can I integrate it easily in my lessons?), if they think that others believe they should use the curriculum (what do my colleagues think?) and the more they are able and skilled to work with the curriculum (can I work with the class material?). Teachers’ experience with the program in turn will influence their intention to use LLL again (continuation) and/or to use the revised LLL (adoption). Their intention is expected to be higher if their attitude toward the program is positive, if they believe other teachers also intend to use the program and if they have the skill and ability to work with the program [23].

In the Netherlands, a study about the adoption and implementation of HIV/AIDS education among 956 Dutch secondary schools showed that teacher’s decision making was most strongly related to highly specific adoption-related beliefs (outcome expectations, subjective norms and self-efficacy) [15]. Similarly, Hoekstra *et al.* [26] investigated teacher’s intentions to use school-based health education programs on self-development and anti-bullying (adoption). Factors that influenced teachers’ intention to use the programs were social norms and outcome expectations concerning the prevention program as well as instrumentality.

### Interactive context

The ‘interactive context’ consists of environmental and organizational conditions in which teachers have to implement sexual education in their school. The interactive context refers to a schools’ formal sexual education policy, governing body support (context), the frequency of collegial interaction about sexual instruction, the extent of use of sexual education curriculum by colleagues (descriptive norm) and the students’ response to the curriculum. Teachers have to teach in collaboration with their colleagues and within the boundaries set by the policy of their school [23]. Curriculum implementation is thus assumed to be facilitated by a clearly stated school policy in the schoolwork plan or their own curriculum work plan [27] and by interactions of teachers with the school management and their colleagues about instructional matters [28]. In case conditions of frequent collegial interaction are not present at a school, perceived behavior of colleagues may operate as a descriptive norm for strengthening teachers’ own implementation decision [29]. Students’ reactions to the curriculum are expected to influence the extent of implementation, with positive reactions resulting in more of the program being used [30].

### Information source

The ‘information source’ refers to support from external consultants, namely the municipal health services (MHS) and additional training for teachers to implement the innovation. In the Netherlands, the MHS is responsible for regional health promotion and supports schools in delivering health education programs. Attending teacher training and receiving external consultation can facilitate adoption and implementation by enhancing teachers’ skills and self-efficacy with regard to sexual education [15]. It has been shown that the provision of pre-implementation training increases the likelihood that teachers will implement the curriculum fully and with integrity [31]. Previous studies found that implementation dose was associated with having received training on that specific curriculum [20, 31–33]. A study examining the extent of school-based tobacco prevention curricula found that trained teachers were more likely to implement and to implement more of the curriculum than untrained teachers [34].

### Demographic variables

Several demographic characteristics of both teachers and their schools may influence adoption and implementation of LLL such as teachers’ gender, age, years of experience with LLL, schools denomination (Catholic, Protestant and Public) and class composition.

### Other variables

To complement the determinant study, several constructs that are not represented in the framework were added, namely what LLL program components teachers use in their lessons (teacher’s manual, student magazine and DVD), the hours they spend on teaching the LLL program and the extent of familiarity with the program prior to using it, as this could influence implementation behavior [35]. Additionally, open-ended questions were included to reveal teachers’ reasons for their intentions to continue using the current LLL program or to adopt the ‘new’ LLL program.

## Method

### Participants and recruitment

A list from the educational publisher of teachers who have ordered the LLL program since 2006 was used to recruit teachers. A questionnaire was sent by post to a total of 610 teachers who are working or have worked with LLL. A total of 130 teachers from 110 schools completed the questionnaire, a response rate of 21.3%. Twenty-five questionnaires were returned due to incorrect addresses or teachers no longer working in those schools. Non-responders got a reminder by post, e-mail and eventually by telephone and were given 2 more weeks to fill out the questionnaire. No official non-response research was conducted due to a shortage of time and schools closing for the summer holiday.

The participating schools were well distributed over the different regions of the Netherlands. Half the schools (50.8%, *N* = 66) had no religious background and a small school size (≤500 students) with 58% (*N* = 73) of teachers having a class compositions of predominantly native students. Of the participating teachers, 104 were female (80%). The mean age was 44 years (SD = 10.4). Years of teaching experience ranged from 1 to 42 years (*M* = 22; SD = 10.3), whereas years of experience teaching sexual education ranged from 1 to 34 (*M* = 8; SD = 6.7) and years of experience with LLL ranged from 1 to 10 (*M* = 4; SD = 2.51). About 94% were teachers of biology and healthcare.

### Procedure

A cross-sectional study of teachers who provide sexual education at secondary vocational schools in the Netherlands, using or having used the LLL program, was conducted. Teachers received an envelope containing an official letter with instructions for filling out the questionnaire and a return-envelope in which they could send back the filled out questionnaire, free of charge. A 10-euro gift voucher as well as the option to participate free-of-charge in the sex education workshops (‘Youngsters, sex and Islam’ and ‘Youngsters, sex and internet’) were offered as reward. Teachers were given 2 weeks to complete and send back the questionnaire. Anonymity and confidentiality were preserved throughout the study.

### Measures

The items included in the questionnaire were based on the scales used by Paulussen *et al.* [15] and Wiefferink *et al.* [23]. Implementation and continuation refer to the current LLL program whereas adoption refers to teacher’s intention to use the ‘new’ LLL that is currently under development.

### Dependent variables

‘Completeness' or ‘extent of use’ of LLL was expressed as the percentage of the program (i.e. learning activities) being implemented. For each of the 22 core learning activities in LLL, teachers were asked if they had completed that activity. The completeness of implementation of the other four activities was not included in the analyses as these were optional. In the end, completeness was calculated for each teacher by adding up all the activities they completed per lesson, dividing them by the total number of activities (maximum 22) and multiplying them by 100.

‘Fidelity' or ‘quality of use’ was measured by asking teachers to indicate, per lesson, how well they followed the instructions in the teacher’s manual (1 = considerably modified it, 2 = slightly modified it and 3 = followed it very closely). The scores per lesson were added up for each teacher and divided by the total number of lessons (6) to produce an average.

‘Continuation’ of current LLL was measured with one item: ‘Do you intend on using the current LLL program next school year for your sexual education lessons?’ (1 = no, certainly not, 5 = yes, certainly). Teachers were asked in an open-ended question to explain their intention level.

‘Adoption’ of the ‘revised’ LLL program was measured with one item: ‘Do you intend on using the revised LLL program in the coming years for your sexual education lessons?’ (1 = no, certainly not, 5 = yes certainly). Adoption has been conceptualized as teacher’s intentions to use the innovation in various other studies [12, 15, 18, 23]. Teachers were asked in an open-ended question to explain their intention level.

### Independent variables

Table I shows an overview of the independent variables, their internal consistency reliabilities, scales and items.

**Table I.** Measures of independent variables

Independent variables	*α*	*N* items	Example items	Scale
**Curriculum-related beliefs**				
Outcome beliefs:	0.91	16	Measured as a weighted result of the teacher’s ‘perceived importance' and ‘perceived feasibility’ (i.e. Σf*i/16)	
Perceived importance of student learning outcomes	0.92	16	‘How important is it to you that your students know what to do when they have an STD or an unexpected pregnancy?’	1 = not important at all, 5 = very important
Perceived feasibility of these outcomes	0.90	16	‘Do you expect to achieve that students can estimate their own risk of contracting an STD or unplanned pregnancy?’	1 = no, not at all, 5 = yes, certainly
Teacher benefits	0.80	7	‘I gained insight in the sexuality experience of youngsters’	1 = strongly disagree, 5 = strongly agree
Instrumentality	0.86	16	‘The time necessary for preparing classroom instruction is acceptable’	1 = completely disagree, 5 = completely agree
Subjective norms	0.81	6	‘Do you think that the following people appreciate you using LLL to provide sexual education?’ (principal, governing body, external consultants/health education experts, students, colleagues teaching the same and colleagues teaching a different subject, parents)	1 = no, certainly not, 5 = yes, certainly
Social support	0.81	4	‘Do you expect support from the following people when implementing LLL?’ (governing body, colleagues teaching the same and different subjects and the parent association)	1 = no, certainly not, 5 = yes, certainly
Self-efficacy	0.89	12	Three skills related domains: (i) use of interactive teaching strategies: ‘I am able to do a condom-use demonstration’, (ii) talking frankly about sexuality: ‘I can openly describe, in the classroom, the different ways of having safe and unsafe sex’ and (iii) using management strategies to create classroom orderliness and safety: ‘I can make the tough behavior of boys discussable so as to not disturb the lesson’	1 = no, certainly not, 5 = yes, certainly
**Interactive context**				
School policy	—	1	‘Is sexual education officially determined as teaching activity in your school?’	0 = no, sexual education is not officially determined, 1 = yes, sexual education is officially determined, 2 = I do not know
Governing body support	—	1	‘Is providing sexual education actively supported and stimulated by the school management?’	1 = no, certainly not, 5 = yes, certainly
Collegial interaction	0.81	4	‘Do you discuss plans about the implementation of new sex education material with colleagues from your department?’	1 = no, certainly not, 5 = yes, certainly
Descriptive norm	—	1	‘Are there other teachers in your school who use or have used LLL?’	0 = no, 1 = yes, 2 = I do not know
Student response	0.79	6	‘Indicate how students generally respond to LLL: interested, shy, comfortable, actively participated, cooperated, enjoyed it’	1 = not at all, 7 = yes, totally
**Information source**				
Teacher training	—	1	‘Did you follow a training in the past four years specifically for the use of the LLL curriculum?’	0 = no, no training was followed, 1 = yes, a training was followed
Contact with MHS	—	1	‘Did you have contact with workers from a local or regional health service (MHS) about the use of LLL in the past four years?’	0 = no, 1 = yes
**Demographic variables**				
Gender	—	1	‘What is your gender?’	0 = female, 1 = male
Age	—	1	‘What is your age’?	
Years of experience with LLL	—	1	‘For how many years have you been using LLL?’	
School size	—	1	‘How many students does your school have?’	1 = maximum 500, 2 = 500–1000, 3 = more than 1000
Class composition	—	1	‘What is the average ethnic composition of students in the class that you teach sexual education?’	1 = predominantly native, 2 = approx. 3/4 native and ¼ foreign, 3 = approx. ½ native ½ foreign, 4 = approx. ¼ native and ¾ foreign, 5 = predominantly foreign
**Other**				
Extent of familiarity	—	1	‘How familiar are you with the LLL program?’	0 = I only bought the program, 1 = I reviewed the program superficially, 2 = I reviewed some parts of the program superficially and other parts thoroughly, 3 = I reviewed the program completely and thoroughly.
Hours spent on LLL	—	1	‘How many hours do you spend on teaching LLL?’	

### Other variables

In relation to LLL, teachers were asked whether they used the DVD, teacher manual and student magazine in the LLL lessons (1 = yes, 0 = no), how familiar they were with the program before using it and how many hours they had spent on teaching the LLL program. ‘Extent of familiarity’ with the program was measured on a 4-point scale from (0) I only bought the program, to (3), I reviewed the program completely and thoroughly.

## Statistical analysis

Descriptive analyses were first conducted to get an overall picture of the research sample. Next, Pearson’s correlation coefficient was used to reveal the correlations between the independent variables and the outcome variables (completeness, fidelity, intention to continue using current LLL and intention to adopt new LLL). Backwards stepwise multiple regression analysis was then used to identify factors associated with these outcome variables. All independent variables were entered at the same time for each outcome variable, respectively. Only variables with significant bivariate associations (*P* < 0.05) were included in the regression equations to understand how much variation in the outcome variables can be predicted by the independent variables. This regression analysis was done separately for each outcome variable. Several factors were dichotomized and included in the regression analysis as dummy variables, namely, school policy, descriptive norm and class composition. Multilevel regression analysis was not necessary because only one or two teachers per school participated in the study. Differences were interpreted as significant when *P* < 0.05.

## Results

Means, standard deviations and inter-correlations of the study measures are shown in Table II. The correlations and explained variances will be discussed per diffusion stage to identify the most important determinants and gain insight into how much of the variance in the diffusions stages can be explained by these determinants.

**Table II.** Descriptive statistics and bivariate correlations between study variables

	*M*	SD	*N*	1	2	3	4	5	6	7	8	9	10	11	12	13	14	15	16	17	18	19	20	21	22	23	24
**Outcome variables**																											
1.Completeness	64.1	25.2	119	1								.															
2.Fidelity	2.1	0.60	119	0.39*	1																						
3. Continuation of current LLL	4.1	1.12	128	0.17*	0.13	1																					
4. Adoption of revised LLL	4.1	0.80	128	0.11	−0.05	0.29*	1																				
**Curriculum-related beliefs**																											
5. Outcome beliefs	17.2	3.21	122	0.11	0.15	0.10	0.12	1						.													
6. Teacher benefits	3.7	0.57	123	0.28*	0.14	0.16	0.29*	0.22*	1																		
7. Instrumentality	3.9	0.47	120	0.27*	0.39*	0.29*	0.21*	0.32*	0.29*	1																	
8. Subjective norm	4.2	0.54	122	0.29*	0.13	0.31*	0.18*	0.19*	0.21*	0.38*	1																
9. Social support	4.4	0.54	122	0.28*	0.10	0.41*	0.19*	0.10	0.26*	0.38*	0.69*	1															
10. Self-efficacy	4.2	0.48	121	0.21*	0.25*	0.28*	0.20	0.49*	0.26*	0.45*	0.32*	0.30*	1														
**Interactive context**																											
11. School policy	1.09	1.0	127	0.06	−0.07	0.17*	0.22	0.15	0.09	0.08	0.14	0.11	0.17	1													
12. Collegial interaction	4.5	0.70	123	0.09	0.21	−0.05	−0.05	−0.02	0.01	0.10	0.22*	0.34*	0.00	0.04	1												
13. Governing body support	4.4	0.85	127	0.03	0.05	0.21*	0.07	0.01	0.21*	0.12	0.13	0.29*	0.19*	−0.18*	0.13	1											
14. Descriptive norm	0.87	0.63	128	0.09	0.10	0.08	0.08	−0.21*	−0.05	0.00	0.03	0.03	−0.15	0.11	0.24*	0.02	1										
15. Student response	5.3	0.74	119	0.16*	0.10	0.37*	0.21	0.30*	0.22*	0.49*	0.28*	0.26*	0.41*	0.13	−0.15	0.16	−0.04	1									
**Information source**																											
16. Contact MHS	0.41	0.49	129	0.17*	0.11	0.10	0.06	0.14	0.13	0.17	0.18*	0.08	0.02	−0.08	−0.02	0.02	−0.05	−0.01	1								
17. Training for LLL	0.38	0.49	129	0.24*	0.23*	0.02	0.07	0.20*	0.11	0.30*	0.14	0.11	0.17	−0.15	0.03	0.05	−0.01	0.05	.39*	1							
**Demographics**																											
18. Age	44.1	10.4	129	0.01	−0.08	−0.08	−0.04	−0.06	−0.11	0.04	−0.06	−0.01	0.08	0.02	−0.00	0.09	0.00	0.07	0.10	0.13	1						
19. Gender	0.20	0.40	130	−0.06	−0.13	−0.00	0.06	−0.11	0.03	−0.08	0.01	−0.05	0.03	−0.04	−0.06	0.05	−0.12	0.05	0.09	−0.04	0.15	1					
20. Years with LLL	4.2	2.51	119	−0.01	−0.16*	0.03	0.06	0.07	0.01	0.00	−0.09	0.07	−0.05	−0.08	0.05	0.05	−0.02	0.05	−0.06	0.15	0.24*	0.12	1				
21. School size	1.77	0.85	129	−0.02	−0.14	−0.05	0.05	0.04	0.07	−0.05	−0.06	−0.13	0.01	−0.12	−0.00	−0.18	0.12	0.05	0.07	0.04	0.03	0.07	0.17	1			
22. Class omposition	2.46	1.57	127	0.03	0.06	0.14	−0.05	0.13	0.14	0.12	−0.02	0.13	0.16	−0.12	0.05	0.10	0.08	0.11	0.19*	0.08	−0.00	0.00	0.06	−0.05	1		
**Other**																											
23. Extent of familiarity	2.47	0.27	129	0.48*	0.31*	0.01	0.10	0.25*	0.13	0.29*	0.18*	0.19*	0.27*	−0.16	0.02	0.15	−0.03	0.29*	0.15	0.33*	0.19*	−0.01	0.04	0.05	−0.10	1	
24. Hours on LLL	6.97	4.64	119	0.45*	0.17	0.27*	0.04	−0.00	0.24*	0.22*	0.18	0.16	0.24*	0.09	−0.01	0.22*	0.12	0.15	0.22*	0.27*	0.04	−0.17	0.07	0.06	0.15	0.32*	1

*P < 0.05, two-tailed.

### Implementation of LLL

Approximately half the teachers report having relational and sexual education somehow formally established in school (55%). More than half of participating teachers had not received a training in sex education at all (58.5%) whereas 38% had received a training specifically for the use of LLL and 3.5% had received a general training in sex education (not LLL specific). The majority of teachers (58.9%) did not have any contact with the MHS in the past 4 years. Those who did have contact predominantly received a training specifically for LLL from the MHS (52.8%).

Teachers generally spent 2–12 h teaching the LLL program, depending on how much time they had available and needed to complete the program. Furthermore, teachers were familiar with LLL; the majority of them had reviewed the program completely and thoroughly before use (59.7%, *n* = 77). Few teachers only superficially reviewed the program (6.2%, *n* = 8) or solely bought it (3.1%, *n* = 4).

### Completeness

On average, teachers implemented 64.1% (ranging 4.5–100%) of the 22 learning activities included in the analyses. Each activity was completed by over 80% of the teachers except homework activities (ranging between 19% and 68%). All components of the LLL program (student magazine, DVD and teacher manual) were used by over 90% of the teachers.

Completeness correlated significantly with numerous factors, namely teacher benefits, instrumentality, subjective norm, social support and self-efficacy, student response, contact with the MHS, following a training specifically for LLL, spending more hours on LLL, fidelity and extent of familiarity with the program (see Table II). Teachers were more likely to use more of the program if they saw benefits in its use for themselves, if they found the program practical to use, if they believed that others appreciate and support their use of LLL to give sexual education and if they believed they are capable of using LLL. They also used more of the program if they receive positive responses from students, are trained by the MHS in the use of LLL and if they were more familiar with the program. Additionally, teachers who spent more teaching hours on LLL and who delivered the program as prescribed use more of the program. The regression analysis revealed that 43.2% of the variance in completeness is explained by these determinants.

### Fidelity

In general, teachers tend to follow the lessons as prescribed or slightly modify their lessons (*M* = 2.1, SD = 0.6). Especially lessons on risks of unsafe sex, negotiating condom use and resisting social pressure to practice unsafe sex were considerably modified compared with the rest.

As shown in Table II, the most important correlates of fidelity are instrumentality, self-efficacy, training for LLL, years of experience and extent of familiarity with LLL. Teachers are more likely to implement the program as prescribed if they find the program to be practical and useful in practice, if they believe they are capable of using LLL, if they follow a training specifically for LLL and if they were more familiar with the program. On the other hand, teachers who have worked with LLL for longer years appear to modify their execution of the program and diverge from the prescription. The regression analysis indicated that 25% of the variance in fidelity is explained by these determinants.

### Continuation of current LLL

The intention level of the group to continue using the current LLL was generally high (*M* = 4.1; SD = 1.12). Factors that appear to significantly predict and explain intention to continue using LLL are instrumentality, subjective norm, social support, self-efficacy, student response, governing body support, school policy, completeness and hours spent on LLL, as shown in Table II. These variables predicted 30.2% of the total variance in intention to continue using the current LLL. Consequently, teachers are more likely to re-use the current LLL program if they find the program practical, believed that others appreciate and support their use of LLL to give sexual education and if they believe they are capable of using LLL. Receiving positive responses from their students, experiencing support from the school management in their implementation of LLL, and established school policy for sex education and taking more time for teaching LLL also predict higher intentions for continuation of LLL.

Explanations for intention level were reported by teachers in the open-ended questions of the questionnaire (*N* = 91). Teachers with higher intentions to continue using the current LLL were more positive about the curriculum (34%) and believed it appealed to students (20.9%). Other teachers are happy to use this program until something better appears on the market (12.1%). Explanations for lower intention levels are that teachers find the material outdated, especially the DVD, and prefer to wait for a new version (13.2%). Some teachers claimed the material no longer appeals to students due to being outdated (6.6%), whereas others found the program lacking modern-day information (5.5%) or found the program too time-consuming (4.4%). Some teachers simply were no longer teaching subjects in which LLL was usually provided (3.3%).

### Adoption of ‘revised' LLL

The intention to use the revised LLL was high (*M* = 4.1; SD = 0.80). Table II shows the predictors of intention to use the new LLL program, namely teacher benefits, instrumentality, subjective norm and social support, followed by intentions to continue using the current LLL version. These factors explained 23% of the variance in intentions to adopt the new LLL.

Explanations for a level of intention to adopt the revised LLL program were reported by teachers in the open-ended questions of the questionnaire (*N* = 74). Teachers with higher intentions to adopt the revised LLL program namely had hopes and expectations that the new program will be an improvement on the previous version (modern, appealing to students and enriched with current issues) (52.7%). Lower intention levels to adopt the new LLL can be explained by teachers’ uncertainty about the content of the new program and a preference to judge for themselves first (47.3%).

## Discussion

The current study has attempted to provide insight into the promoting and inhibiting factors of adoption, implementation and continuation of the school-based sex-education program, Long Live Love. Different factors influence each stage in the diffusion process and understanding factors influencing each of these stages is essential for successful implementation [30]. The different stages are however dependent on one another and complementary.

A positive result of this study is that most of the LLL program is delivered and that teachers generally do this with relative integrity. On average, teachers carried out approximately two-thirds of the activities to be implemented from the program and delivered the lessons as prescribed or only modified them slightly. This is a promising result as several studies indicate that programs are frequently modified during implementation [36–38] and teachers do not always implement programs according to specific guidelines [30]. Intentions to continue using the current LLL were relatively high as was the intention to adopt the revised LLL.

Teachers’ curriculum-related beliefs were found to be important for all stages of the diffusion process. ‘Implementation’ (completeness and fidelity) was especially related to following a training specifically for LLL, greater instrumentality of the program, higher self-efficacy and greater familiarity with the program. Teachers who followed the guidelines of the LLL program more closely (fidelity) also completed more of the program (completeness). These findings are similar to a process evaluation study of a school-based adolescent sexual health intervention in rural Tanzania, where teachers delivered the program to primary school students with remarkable integrity and this fidelity was enhanced by a training course [39]. Teacher curriculum-related beliefs and information source variables are therefore essential for implementation [40, 41].

Fidelity was however hindered in our study when teachers had more years of experience with LLL. Several studies indicate that programs are frequently modified in the process of implementation [30, 36–38]. Years of experience with a program may lead to reinvention of it by the user to accommodate the changing circumstances in schools and diversity in composition of classrooms (gender, ethnicity or sexual experience of students) in time [36–38].

‘Continued use’ of LLL was positively related to interactive context variables (student response, governing body support and having a school policy for sex education formally established) and curriculum-related beliefs (instrumentality, subjective norms, social support and self-efficacy). Furthermore, the more of the curriculum the teacher completed and the more hours they spent on LLL, the higher the intention to continue using LLL. Information source variables did not correlate with continued use.

‘Adoption’ of the revised LLL was predominantly related to curriculum-related beliefs, namely teacher benefits, instrumentality, subjective norm and social support. Also, teachers who had a higher intention to continue using LLL were also more likely to adopt the new LLL. Information source variables and interactive context variables did not correlate with adoption. Self-efficacy correlated with adoption but not significantly. This runs counter to the findings of other studies, in which self-efficacy appeared to be a dominant predictor of teachers’ decision making on innovations [23]. Perhaps with a higher power, self-efficacy would have been found to be significant. One possible explanation why self-efficacy was not a dominant predictor of adoption might be based on the correlations between outcome beliefs, instrumentality subjective norms and teacher benefits on the one hand, and self-efficacy on the other hand. This would correspond with the theory of Bandura that self-efficacy predicts outcome beliefs and other cognitions and that these factors in turn predict behavior [24]. Another explanation might be that teachers’ efficacy is less dominant during the stage of adoption than during implementation [30].

To date, only one study is comparable to the current one: the Sexual Health and Relationships: Safe, Happy and Responsible (SHARE) program in Scotland [9]. The SHARE study examined factors that impeded or facilitated the implementation of a teacher-delivered sex-education program for youth (13–15 years old). Results showed that fidelity was aided by intensive teacher training, classroom compatibility and senior management support while it was hindered by competition for curriculum time, brevity of lessons and teachers’ limited experience and ability in use of role-play [9]. Paulussen [15] found that teacher’s adoption and implementation behaviour of HIV programs were most strongly related to teacher beliefs (attitudes, social influences and self-efficacy), as this study also suggests. Generally, teachers will teach best in areas for which they are best prepared, have effective materials and techniques, and for which they receive recognition and support from school administrators and colleagues [34].

Results of this study show that adoption is predominantly related to individual level factors, whereas implementation and continuation are also influenced by external factors, namely information source variables and the interactive context, respectively. Teacher training is an information source variable that is especially important in stimulating complete and correct use of LLL. It has been identified previously as a major determinant of success in the implementation of school-based programs [42, 43]. Pre-implementation training has been found to increase the integrity with which teachers implement a curriculum [37, 44–46] because it enhances teachers’ skills that are relevant to the intervention program [19]. In the Netherlands, teacher training in sexual health promotion is provided by the MHS.

Continued use of LLL is largely dependent on conditions that enable structural embedding of LLL, namely a supportive school management and school policy formally establishing sexual education in the school. Also, observing positive student responses reinforces implementation behavior of teachers [12]. In South Africa, ongoing engagement and support of teachers were also found to play an important role in their ownership of an AIDS prevention curriculum and partially explained continued use of the program [47]. Support motivates teachers to implement the program, and in doing so correctly, they are likely to experience further success in changing their students’ behavior in the classroom, which in turn leads to continued program use [19]. School policy has also been found to be essential in contributing to a successful diffusion process [11, 12, 27].

The study had some limitations. Due to the cross-sectional design of this study, conducted at one moment in time, it is impossible to determine whether the teacher curriculum-related beliefs precede teacher’s implementation behavior or if they are a result of it. No present conclusions can be drawn about causality, only associations, unless a longitudinal study is conducted. Also, the same measures were used to predict implementation and continuation of the current LLL as adoption of the ‘revised’ LLL, which could limit interpretations of the adoption results. Past experiences with a program have however been found to predict future use [12, 19, 20, 21].

Additional methodological limitations of the study are self-reports by teachers, a self-selected sample, limited measurements on the outcome measures of adoption and continuation with a single item and lack of assessment of student outcomes. An effectiveness study was however conducted in 2001, where LLL was found to have positive outcomes for students, proving that it is a worthy program [14]. Process evaluation remains essential for examining the quality and extent of program implementation and understanding the effects of interventions [48]. Observation of fidelity and rapport would have further validated the results of this study.

With this study, we hope to share lessons for successful implementation in the school setting. The results reveal that each stage of the diffusion process is influenced by different kinds of factors. This implies that it is necessary to consider all three stages when planning and evaluating the implementation of interventions. It also implies that different strategies are needed to enhance adoption, implementation and continuation of an innovation as the Diffusion Theory suggests [16]. To enhance adoption, the focus should be predominantly on teacher curriculum-related beliefs, presenting the personal benefits of using the intervention, providing support for use of it and developing a practical and easy-to-use intervention. Implementation is further supported by equipping teachers with knowledge and skills through training to promote quantity and quality of implementation. Continuation is attained by a supportive school policy and climate of personal support for teachers [11]. The implication for health education is that in addition to addressing more traditional factors such as training, and teacher beliefs, the program planners should also consider the climate of the organization [49]. These broader contextual factors may support or inhibit teacher’s efforts at program implementation [19].

Much needed insight has been provided for the facilitating and inhibiting factors influencing the different stages of the implementation process of a school-based sex-education program, LLL. This information becomes especially relevant in the field of health promotion intervention development, where the importance of implementation is being increasingly acknowledged [5]. Understanding the determinants of the implementation process of LLL will not only benefit the extent and quality of implementation of the future updated LLL program or provide inspiration for the systematic development of an implementation strategy but also provides possible explanations for effectiveness of such curricula and why these may succeed or fail when conducted in a real-world setting. The suggested recommendations may lead to improved implementation of school-based sex-education programs internationally and locally, contributing significantly to a better-equipped and knowledgeable youth concerning sexuality and relations. Long live love!
